# Virtual Reality Cue Refusal Video Game for Alcohol and Cigarette Recovery Support: Summative Study

**DOI:** 10.2196/games.9231

**Published:** 2018-04-16

**Authors:** Mary Metcalf, Karen Rossie, Katie Stokes, Christina Tallman, Bradley Tanner

**Affiliations:** ^1^ Clinical Tools, Inc Chapel Hill, NC United States

**Keywords:** addiction treatment, Kinect, serious games, motion control games, virtual reality

## Abstract

**Background:**

New technologies such as virtual reality, augmented reality, and video games hold promise to support and enhance individuals in addiction treatment and recovery. Quitting or decreasing cigarette or alcohol use can lead to significant health improvements for individuals, decreasing heart disease risk and cancer risks (for both nicotine and alcohol use), among others. However, remaining in recovery from use is a significant challenge for most individuals.

**Objective:**

We developed and assessed the Take Control game, a partially immersive Kinect for Windows platform game that allows users to counter substance cues through active movements (hitting, kicking, etc).

**Methods:**

Formative analysis during phase I and phase II guided development. We conducted a small wait-list control trial using a quasi-random sampling technique (systematic) with 61 participants in recovery from addiction to alcohol or tobacco. Participants used the game 3 times and reported on substance use, cravings, satisfaction with the game experience, self-efficacy related to recovery, and side effects from exposure to a virtual reality intervention and substance cues.

**Results:**

Participants found the game engaging and fun and felt playing the game would support recovery efforts. On average, reported substance use decreased for participants during the intervention period. Participants in recovery for alcohol use saw more benefit than those in recovery for tobacco use, with a statistically significant increase in self-efficacy, attitude, and behavior during the intervention. Side effects from the use of a virtual reality intervention were minor and decreased over time; cravings and side effects also decreased during the study.

**Conclusions:**

The preliminary results suggest the intervention holds promise as an adjunct to standard treatment for those in recovery, particularly from alcohol use.

## Introduction

### Theoretical Basis for the Game Intervention

The Take Control recovery support game uses several familiar and well-researched therapies to improve player treatment outcomes for addiction. Cue exposure therapy (CET) is a commonly used method in substance abuse treatment [1-6]. Traditionally, CET is performed with pictures of a substance, the actual substance itself, or even its scent [2]. The patient is repeatedly exposed to cues and stimuli and encouraged to ignore the craving response or use a coping response [7-10]. In keeping with the theory that the treatment effect is due to practicing a healthier response to a cue, the player in our game is repeatedly exposed to an image of a substance, and rather than responding with use, the player is trained to substitute a more dynamic, adaptive response. They must react appropriately (destroy the substance) in order to advance in the game.

Another way to explain the effect of this game is counter-conditioning. The unwanted behavior of responding to the cue to use a substance is being replaced with a positive action, and the new behavior is rewarded [11]. The game supports the rehearsal of the positive action of actively refusing a substance when it is presented and offers a reward to reinforce the more positive response in the form of success in the game.

The effectiveness of this game’s approach to substance abuse treatment also might be partially explained by the extinction response. Extinction therapy aims to reduce a patient’s conditioned response to a substance by repeated exposure without reinforcers in order to dull the craving response over time [12,13]. In our game, the patient is repeatedly exposed visually to the substance without receiving the reinforcing effect of the substance, which may produce some extinction effects. Creating new memories will overlap former memories, thus extinguishing old habits and responses [13]. Our game will allow players to use movement to form new, more adaptive associations with the substances.

Virtual reality therapy (VRT) uses virtual environments to expose patients to stimuli in a safe and controlled manner, such as with phobias or posttraumatic stress disorder [14]. In VRT for addiction treatment, the stimulus is the substance of the patient’s addiction [5,7,9,10,15,16]. A VR video game has distinct advantages over other exposure methods (eg, pictures produced by a counselor). Being in a VR environment allows the player to feel more immersed in the game, resulting in greater involvement and translation into real-life actions [5]. The game also addresses the need for a safe environment to practice refusal skills, as seen in coping skills training [5,17].

Exercise, which has been shown to aid in recovery from substance use disorders [18] and reduce comorbid factors that hinder overall health and wellbeing [19,20], is another factor that may mediate the game’s effect. Free movement is possible with our game because Kinect (the system) is not hindered by a controller, cords, or bulky head gear.

Cognitive behavioral therapy (CBT) has extensive research backing its effectiveness in addiction treatment [21,22]. One important interpersonal component of CBT is refusal skill practice. Patients learn how to respond rapidly, maintain eye contact, and give a clear “no” when offered drugs [23]. In our proposed game, players will practice refusal skills (such as verbally saying “no” and physically turning one’s back) when offered a substance by a character.

In the specific field of technology-based interventions for substance abuse, we did not identify any similar games to Take Control. However, there was a related study conducted by Girard et al [24] showing that 4 sessions performing behaviors incompatible with smoking cigarettes (crushing virtual cigarettes) within a virtual environment were more efficacious for smoking cessation than a similar game in which patients found and crushed virtual balls. The mechanism of this treatment in the study was not well understood, but we surmised that such virtual practice in a game environment may be uniquely helpful because it can deliver a large dose of alternative practice in a manner that people not only tolerate but enjoy. The fact that a short duration gaming experience in Girard et al [24] could improve outcomes in comparison with a placebo control suggests that games that involve the body in alternative practice may hold promise for treating addiction.

### Game Design

The Take Control recovery support game was developed for use with the Kinect motion sensor camera and device available with Xbox One and Windows operating systems.

**Figure 1:**
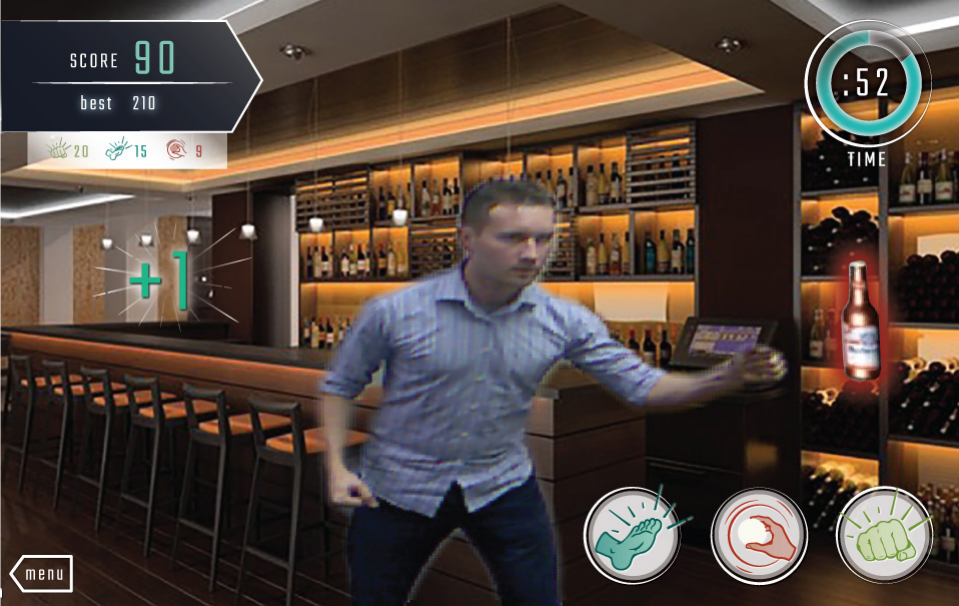
A staff developer swipes away a beer bottle during game play.

Users hit or kick away cue images as they fly toward the user, as seen in Figure 1. If a user successfully hits the image, it explodes and the user gains in-game points. If an image hits the user or flies off the screen without being exploded, there is no negative consequence to the user’s score.

**Figure 2:**
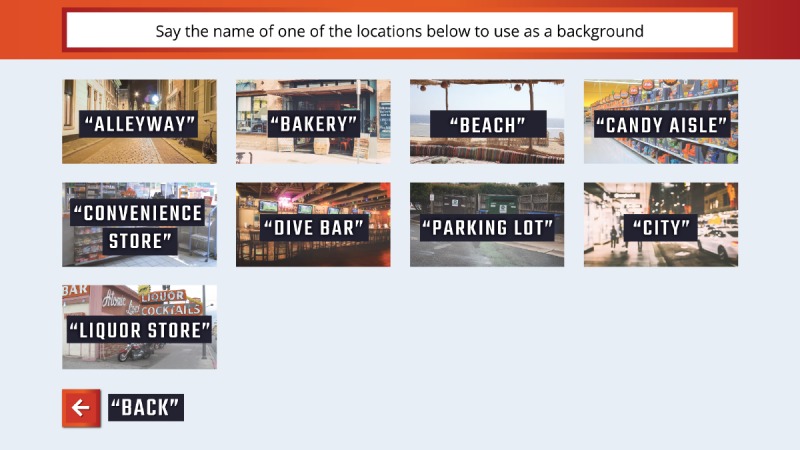
Players can select specific backgrounds for the game.

Users choose a background image, like the one in Figure 2, from a menu using voice or mouse controls and then choose 1 cue item to reject per round. Users were encouraged to focus on 1 substance but could change items between rounds. Users could replay the game using different backgrounds or cues as often as desired.

**Figure 3:**
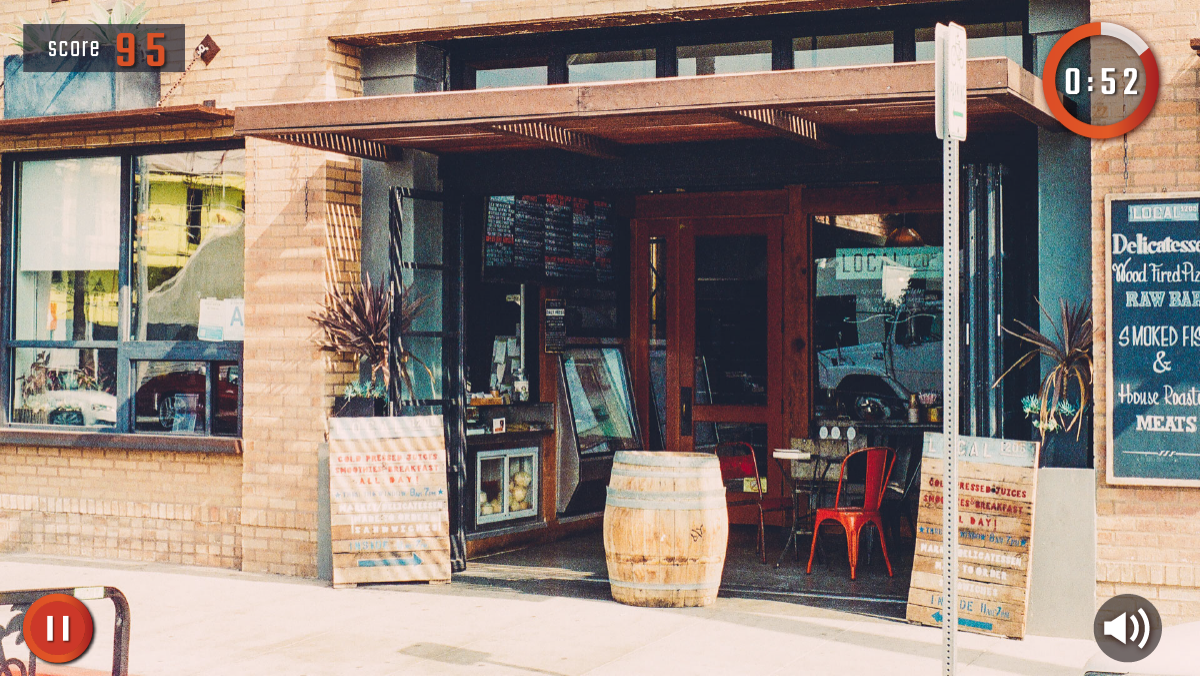
Game backgrounds include realistic photos.

**Figure 4:**
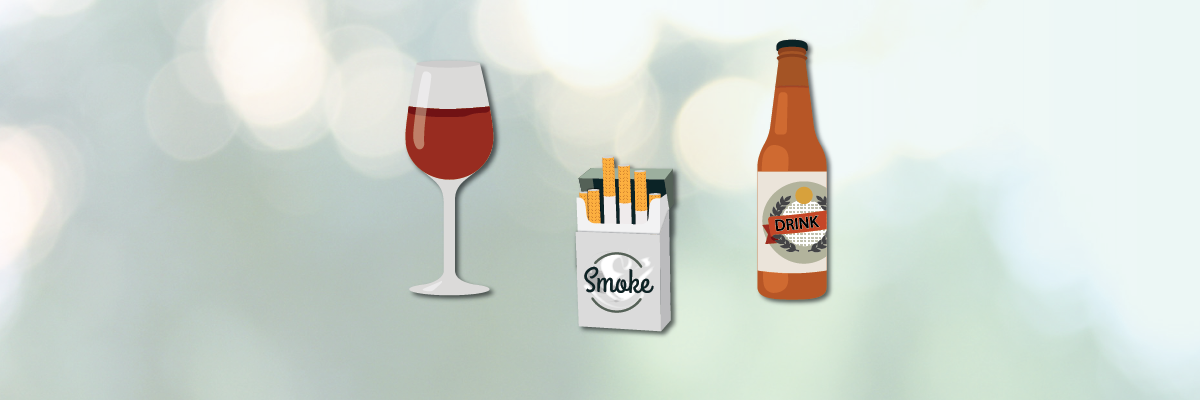
Substance images were drawn to be less specific as preferred by the target audience.

The game includes photo realistic backgrounds, seen in Figure 3, but drawn substance images, seen in Figure 4. During formative studies with target audience members, it was determined that photo realistic images of cue items (cigarettes, beer bottles, etc) were not preferred since users felt that such images were too specific, and thus made the experience less relevant to them individually. There were also reports from formative testers that they believed realistic looking cues might trigger cravings, while illustrations would be less likely to do so.

### Objectives

This study considers how a lower cost, easily accessible video game could be used to support recovery treatment or individual self-efficacy, attitude, and behavior. The goal is to support users in practicing refusal skills and increase self-efficacy by denying trigger or cue items in the nonthreatening environment of the video game. Using realistic backgrounds, users can hit or kick trigger items that fly toward them. Hit items explode, and users gain points.

Primary outcome measures were an increase in reported self-efficacy, attitude, and behavior, a decrease or lack of increase in craving after having seen the trigger items, and satisfaction with the game experience. Self-reported data on continued recovery status were also assessed.

The primary goal of the study was to increase user self-efficacy, attitude, and behavior by allowing the user to practice refusing trigger items in the game context.

## Methods

### Participants

The study was reviewed and approved by the Clinical Tools Inc Institutional Review Board. Participants were healthy adult volunteers who self-identified as having recently quit using cigarettes, tobacco, or alcohol. We did not collect data on coaddictions. Participants were recruited through advertisements in a local weekly news circular, on the Internet (Raleigh Craigslist), flyers placed in local public places (community centers, outside grocery stores), and via word of mouth.

Interested volunteers completed a short, open online survey that reviewed eligibility requirements (aged over 18 years, recent quitting, lack of mobility issues that would prohibit game use, fluent in English). The survey contained an informed consent section with study purpose, methods and procedures, confidentiality, benefits and inconveniences, precautions and risks, and survey submissions were limited via IP addresses. Participants also had to be able to travel to a game setup location. For most participants, this was a small office near a local church building, on the local free bus line, with free parking available. Participants received a gift card to a national store with multiple locations in the area at each of the 4 possible sessions. Sessions typically lasted more than 15 minutes but less than 30 minutes. Participants were asked to play 8 rounds (60 seconds per round) of the game.

Use of the video game was private; research staff were available to assist with any technical difficulties or usability question outside of the testing room, but staff did observe use of the game through a window (where players could not see them) to allow users to behave naturally and not feel judged for their ability to play the game. A computer log documented use time and score, and this was associated with the participant number. The study version incorporated the Kinect for Windows software and ran on a personal computer with a large monitor to facilitate viewing of the game.

**Case and wait-list control group schedules.**

Case:

- Preassessment and Take Control game session 1
- Take Control game session 2 and assessment
- Take Control game session 3 and assessment
- One- to 2-week interval
- Follow-up assessment

Wait-list control:

- Preassessment
- Two-week interval—no assessment
- Preassessment and Take Control game session 1
- Take Control game session 2 and assessment
- Take Control game session 3 and assessment

### Data Collection

The study was a quasi-experimental, stratified, wait-list control trial using a convenience sample due to time limitations.

Participants in each group had the opportunity to play the game at the Clinical Tools office 3 times, with 7 to 12 days in between uses. Case group participants were asked to complete a follow-up set of measures 1 to 2 weeks after the final game use. Control group users completed a baseline set of instruments and then after at least a 2-week wait period repeated the baseline measures and then used the game 3 times.

Thus, each group had a total of 4 assessment interactions and 3 game play interactions (see Textbox 1). Assessments were mostly Likert-style questions except for the 7-Day Timeline (fill-in-the-blank), Side Effects (multiple choice), and Stages of Change (multiple choice). A random number was assigned to participants and the information kept in a locked location. Researchers used this number to log participants into the surveys associated with specific session numbers to keep the participant’s multiple sessions linked.

Data collected for this study were sent to University of North Carolina at Chapel Hill, where a doctoral student in statistics analyzed the data.

## Results

### Participants

A total of 76 participants were enrolled in the summative study. A total of 7 case participants were withdrawn from the analysis: 2 case participants were withdrawn due to inconsistent data and 5 were withdrawn due to not completing the study. A total of 8 control participants were withdrawn from the study, all due to not completing the study. A total of 61 participants were included in the analysis. There were 28 females (1 Asian, 10 African American, 13 white, 1 other, 2 multiracial, 1 prefer not to answer) and 32 males (1 Asian, 14 African American, 15 white, 1 other, 1 prefer not to answer); 1 participant was unknown (chose prefer not to answer for both gender and race categories).

### Data Collection

#### Quantitative Results—Substance Use

The 7-Day Timeline instrument allowed the participant to report any substance they had used the week prior to playing the game. In Table 1, percentages are reported based on a starting point of 100% for those reporting use of a substance in session 1. After playing the game twice, case participants who reported using a substance on the first 7-Day Timeline (n=17) had an average drop to 38% of what they had been using at baseline. Of those who reported some substance use at baseline, 5 out of 17 (29%) stopped using altogether (0% use) after 2 weeks of participation. Control participants who reported using a substance on the first 7-Day Timeline had an average substance use increase from 100% to 110% during the 2-week period prior to playing the game. As shown in Table 2, average substance use increased between the last game play (session 3) and 1-week follow-up (session 4) for those who completed the optional fourth session (15/17). Therefore, they went from 100% at week 1 down to 38% at week 3, and back up (average session 3) to 52% at week 4 (average session 4). Overall, there was improvement from session 1 to session 4 of about 50%.

At the follow-up point for the 7-Day Timeline, a third of those who filled out the follow-up survey had reached 0% use by the last game play session and maintained abstinence. However, 27% of participants (4/15) increased substance use after completing the study. One participant, who had reported 0% substance use at all 3 game play sessions, reported using again at follow-up.

**Table 1:** Change in substance use after 2 weeks (percentage^a^ based on first reported use).

Characteristic	Control without game (n=11)	Case with game (n=17)
Average change in substance use compared to initial 100%, %^a^	110	38
Number who quit, n	0	5
Number who restarted, n	2	1

^a^Percentage reflects the amount of substance use increase.

**Table 2:** Substance use in case group (n=15). N/A: not applicable.

Use for participants who completed session 4 from 7-Day Timeline		Session 1	Session 3	Session 4
**Participants still using their substance**				
	Used substance, %	100.00	37.74	52.37
	Increased since previous session, n	N/A	2	4
	Decreased since previous session, n	N/A	7	2
	Use stayed the same, n	N/A	1	3
	Decreased and stayed at 0% use, n		5	6
	Total, n	15	15	15
**Participants with no substance use or those at 0%, n**		15	15	14
	Restarted	N/A	N/A	1

#### Quantitative Results—Self-Efficacy, Attitude, Behavior, or Intended Behavior

##### Self-Efficacy Results

In general, participants reported an increase in self-efficacy after spending 3 weeks playing the game. Table 3 displays results for each self-efficacy question and shows a growth trend for self-efficacy between sessions 1 and 3, followed by a decrease by the 1-week follow-up session 4. The difference between sessions 3 and 4 showed the most decrease in patient self-efficacy. However, on the fourth week, after not coming back and playing the game, participant average self-efficacy rating drops off. We found differences between session 3 and session 4 values in that most of the participants (21/29) had a decreased self-efficacy score or remained the same. Only 8 participants had increased self-efficacy at session 4, a week after the last time they played the game.

##### Intended Behavior Results

Several individual measures improved for case intended behavior from baseline (week 1) through 1-week follow-up (week 4). Case participants, on average, showed an increase from baseline to 1-week follow-up in their ratings on a 5-point Likert-type scale of their intentions to use health care (0.61 points), resources (0.24 points), and support groups (0.18 points) to assist with their substance use issues. All other intended behaviors measured showed a slight downward trend from baseline to follow-up (Table 3).

##### Attitude Results

Scores on attitude questions, which focused on self-responsibility to use help, started fairly low at week 1 (average 3.88), rose slightly by week 3 (average 3.99), and fell even below baseline by 1-week follow-up (average 3.67).

#### Quantitative Results—Alcohol versus Tobacco

Self-reported, self-efficacy, attitude, and behavior scores that were collected via Likert-style surveys at the beginning of the first game play session, and after the third, or last, session of game play for both case and control were analyzed by the substance used. Participants who had selected alcohol as their problem substance showed improvement in scores from an average of 4.19 at baseline (game play 1) to 4.31 at the third session (game play 3)—an increase of 0.11 (2-tailed *t* test, *P*=.09; see Multimedia Appendix 1). When participants who were still using alcohol at baseline were considered separately (11/26), the increase in scores over the 3 weeks was significant (going from 4.04 to 4.28, *P*=.03). In contrast, those who chose tobacco howed a slight decrease in self-efficacy scores of 0.02 points in the 2 weeks. Tobacco substance users had a decrease in mean self-efficacy score of 0.07.

The rate of participants continuing substance use after entering the study decreased for both alcohol and tobacco users (Table 4). Participants with alcohol substance use decreased their amount of substance used by 75%, whereas tobacco substance users only decreased their substance use by 4%.

**Table 3:** Case player self-assessment scores (5-point Likert-type scale; n=29).

Characteristics		Session 1 (Baseline)	Session 3	Session 1 to 3 (Difference)	Session 4 (1-week follow-up)
**Self-efficacy—I currently feel that:**					
	I am happy with how far I have come in my substance use treatment and recovery	4.07	4.17	0.10	4.14
	I feel good about my future regarding substance use abstinence	4.21	4.34	0.13	4.03
	I am confident in my ability to overcome my substance use issue	4.07	4.38	0.31	4.07
	I am confident in my ability to refuse the use of problematic substances (alcohol/drugs/tobacco)	3.93	4.07	0.14	3.79
	Average self-efficacy	4.07	4.24	0.17	4.01
**Attitude—It is my responsibility to take control of my substance use issues by:**					
	Using the help of support channels	4.00	4.07	0.07	3.86
	Using the help of health care professionals	3.41	3.72	0.31	3.45
	Using the help of friends	4.24	4.17	–0.07	3.97
	Average attitude	3.88	3.99	0.10	3.67
Behavior—I intend to quit using problematic substances (alcohol/drugs/tobacco)		4.27	4.00	–0.27	3.93
I intend to reduce my use of problematic substances (alcohol/drugs/tobacco)		4.56	4.43	–0.13	4.15
**Behavior—I intend to use or continue using the help of:**					
	Health care to assist with my substance use issues	2.96	3.57	0.61	3.41
	Friends to assist with my substance use issues	4.07	4.03	–0.04	3.90
	Family to assist with my substance use issues	3.43	3.61	0.18	3.34
	Support groups to assist with my substance use issues	3.54	3.50	–0.04	3.61
	Resources to assist with my substance use issues	3.79	4.03	0.24	3.93
**Behavior—I intend to seek out and participate in:**					
	Healthy lifestyle behaviors such as eating healthily	4.31	4.48	0.17	4.10
	Healthy lifestyle behaviors such as exercising	4.38	4.48	0.10	4.21
	Healthy lifestyle behaviors such as socializing	4.41	4.48	0.07	4.03
	Healthy lifestyle behaviors such as hobbies	4.34	4.62	0.32	4.24
	Average behavior or intended behavior	4.00	4.11	0.11	3.89

**Table 4:** Average change in substance use for participants who started the study using a substance within the past week (n=24).

Substance type	Session 1 attendance, n (%)	Session 3 attendance, n (%)
Tobacco	13 (100)	12 (96)
Alcohol	11 (100)	7 (25)

#### Quantitative Results—Satisfaction

The trend, on average, was that participant satisfaction with the game was positive, with scores averaging between 3.34 and 4.25 (neutral and agreement) on a 5-point Likert-type scale in response to 5 satisfaction questions (see Table 5). The average agreement decreased slightly by the end of the study, perhaps because of the decrease in novelty or instrument fatigue. Participants agreed with all satisfaction statements at the end of the study, however.

#### Qualitative Results

A total of 48 participants offered additional comments in the surveys as well as in an unstructured interview after playing the game. The positive and negative comments were divided into more general and specific comments, and the game design suggestions were recorded. Of the 76 unique comments, 50% (38/75) were positive about the game and playing the game, 9% (7/75) were negative, and 41% (31/75) were neutral or involved game design suggestions for the future (such as changes to fonts, scoring, additional backgrounds or images). Many of the positive comments (21/38) included evaluative statements like, “Fun,” “Cool,” and “Liked it.” The negative comments comprised a mixture of skepticism about the game efficacy, stress inducement, soreness, or general dislike.

**Table 5:** Game satisfaction scores (5-point Likert-type scale). N/A: not applicable.

Game satisfaction		Session 1 (n=52)	Session 2 (n=52)	Session 3 (n=52)	Session 4 (1-week follow-up; n=29)
**Game focus—I feel that:**					
	The game was fun.	4.43	4.48	4.37	4.10
	This game was engaging.	4.58	4.48	4.48	4.17
	Based on my experience, I would recommend this game to other patients in treatment for substance use problems.	3.88	3.98	4.00	3.67
	Based on my experience, this game will aid in my substance use treatment and recovery.	3.45	3.55	3.81	3.53
	Overall, this game will be a useful substance use treatment and recovery tool.	3.78	3.83	4.12	3.70
**Game feedback—The game seems like it will help with my treatment in terms of:**					
	Relapse prevention	3.41	3.40	3.65	N/A
	Seeking help	3.22	3.10	3.38	N/A
	Sticking to treatment	3.68	3.60	3.88	N/A
	Better long-term outcomes	3.76	3.65	3.69	N/A
	Higher quality of life	3.61	3.70	3.71	N/A

## Discussion

### Quantitative Results

There are several important results of this brief study. First, that participation in the study and use of the game seem to support abstinence from substance use based on the 7-Day Timeline reports when game users are compared to participants in the control group. The 7-Day Timeline instrument reported decreased or maintenance of no substance use at a poststudy follow up for most of the 29 participants in the game use group. That is, substance use decreased or remained the same for most users, although more for alcohol users than for tobacco users.

This suggests that participation in the study did support abstinence from substance use and that the effect might be stronger for alcohol use than tobacco use. Additional research is needed to determine if this effect is due to participation in a study or the intervention.

A second finding is that there were fewer positive results seen in substance use during the wait-list control period for those participants. In other words, there was a general increase in use, not decrease while controls waited. This suggests that being in a study and knowing that they would have to report on their substance use did not change their baseline behavior. This result strengthens the argument that it was use of the game that decreased substance use for participants, rather than participation in the study. Future research could examine this finding further.

A third finding is that scores for self-efficacy, attitude, and intended behavior went up significantly from baseline to week 3 (*P*=.03) for patients still using alcohol at baseline. This, together with the greater decrease in substance use for the group that selected alcohol as the substance to work on, suggests that the game benefits may be greater with respect to alcohol use.

Finally, it is noteworthy that few participants reported an increase in use of their substance. This was a concern due to the possibility, as seen in CET modalities, that exposing users to cues for substance use can have a triggering effect and thus have the potential to undermine recovery. This did not happen often, which is encouraging and a necessary result for further research into the use of game- or electronic-based CET adjunctive technologies.

### Participant Satisfaction, Self-Efficacy, and Behavior Key Findings

Secondary findings revolve around participant enthusiasm for the game experience and impact the potential of the game as a supportive product in the future. Participants found the game engaging and fun.

Additionally, participants felt playing the game during recovery would help with relapse prevention and related behaviors. Agreement with this was highest after session 3.

There was a slight but intriguing difference in results for those who reported recovery for alcohol use as their primary goal versus those who chose recovery from tobacco use. Specifically, self-efficacy increased for those in recovery for alcohol use, but there was a minor, not statistically significant decrease in self-efficacy for former tobacco users. Differences between these 2 groups could be an area of further research, as misuse of both substances is harmful to health in the US population. Another improvement seen from pre- to postintervention follow-up was an increased intention to use health care, resources, and support groups.

### Limitations

There are limitations worth noting in this study. The study population was small and was a convenience sample of participants who were interested in maintaining their status in recovery, and thus they may not be typical of all individuals having substance use problems. Second, time was constrained, which impacted how often participants could use the game. In an ideal setting, the game experience would be available to participants more frequently, and a dose-response investigation could be conducted.

Kinect is a kinesthetic game and requires a minimum level of physical ability to move arms or legs, and this limits the reach of the game. A participant was able to use the game from a wheelchair in early testing, but more modifications are needed to effectively reach a mobility-limited population. Also, in terms of the physicality of the game, we believe that the possible aggressiveness of the game is balanced by the positive interactions of taking control of one’s environment; however, a psychological professional would need to evaluate whether this game is appropriate for individuals with aggressive tendencies.

Given this was a short-term feasibility study, long-term studies would need to be conducted to address the complexities of rehabilitation from various addictions.

Finally, Microsoft is no longer actively developing Kinect applications; although current versions of the Xbox One continue to support use (as of August 2017). Thus, future versions of the game should explore additional platforms while maintaining the kinesthetic element of game play and explore how this impacts results.

### Conclusions

This study indicates that a serious game–based intervention has potential to be a useful part of recovery efforts for individuals seeking to maintain abstinence from alcohol or tobacco misuse or use. The use of a kinesthetic game based in a cue refusal theory framework-based intervention could prove a valuable adjunct to therapy in the future. Games have the ability to reach and engage a significant audience segment, and the use of an individually tailored game could expand potential treatment experiences.

## Appendix

Multimedia Appendix 1 Self-efficacy, attitude, and intended behavior scores for case and control participants who completed 3 game play sessions (N=50).

## Abbreviations

CBT: cognitive behavior therapy
CET: cue exposure therapy
SBIR: Small Business Innovation Research
VRT: virtual reality therapy
