# Supplementary material for: Virtual Reality Cue Refusal Video Game for Alcohol and Cigarette Recovery Support: Summative Study
Source: JMIR Serious Games. 2018 Apr 16;6(2):e7. doi: 10.2196/games.9231 (PMC5928331; doi:10.2196/games.9231)
Supplement: Multimedia Appendix 1 [file games_v6i2e7_app1.pdf]

Multimedia Appendix 1. Self-Efficacy, Attitude, and Intended Behavior Scores for Case and Control Participants Who Completed 3 Gameplay Sessions (N=50).

Average Self-Efficacy, Attitude, Intended Behavior score for Case and Control Participants Who Completed 3 Gameplay Sessions	Number of Participants(N)	Gameplay 1	Gameplay 3	Difference
Tobacco	24	3.99	3.97	-0.02
	P (T<=t) one-tail	t Critical one-tail	P (T<=t) two-tail	t Critical two-tail
	0.41	1.71	0.83	2.07
Alcohol	26	4.19	4.31	0.11
	P (T<=t) one-tail	t Critical one-tail	P (T<=t) two-tail	t Critical two-tail
	0.04	1.71	0.09	2.06
